# Results from transcranial Doppler examination on children and adolescents with sickle cell disease and correlation between the time-averaged maximum mean velocity and hematological characteristics: a cross-sectional analytical study

**DOI:** 10.1590/S1516-31802011000300003

**Published:** 2011-05-05

**Authors:** Mary Hokazono, Gisele Sampaio Silva, Edina Mariko Koga Silva, Josefina Aparecida Pellegrini Braga

**Affiliations:** I MD, MSc. Pediatrician, Department of Pediatrics, Universidade Federal de São Paulo (Unifesp), São Paulo, Brazil.; II MD, PhD. Neurologist, Department of Neurology, Universidade Federal de São Paulo (Unifesp), São Paulo, Brazil.; III MD, PhD. Adjunct Professor of Evidence-Based Medicine and Emergency Medicine, Universidade Federal de São Paulo (Unifesp), São Paulo, Brazil.; IV MD, PhD. Adjunct Professor, Discipline of Pediatric Specialties, Division of Pediatric Hematology, Department of Pediatrics, Universidade Federal de São Paulo (Unifesp), São Paulo, Brazil.

**Keywords:** Ultrasonography, Doppler, transcranial, Anemia, sickle cell, Hemoglobin SC disease, Stroke, Child, Adolescent, Ultrassonografia Doppler transcraniana, Anemia falciforme, Doença da hemoglobina SC, Acidente cerebral vascular, Criança, Adolescente

## Abstract

**CONTEXT AND OBJECTIVE:**

Transcranial Doppler (TCD) detects stroke risk among children with sickle cell anemia (SCA). Our aim was to evaluate TCD findings in patients with different sickle cell disease (SCD) genotypes and correlate the time-averaged maximum mean (TAMM) velocity with hematological characteristics.

**DESIGN AND SETTING:**

Cross-sectional analytical study in the Pediatric Hematology sector, Universidade Federal de São Paulo.

**METHODS:**

85 SCD patients of both sexes, aged 2-18 years, were evaluated, divided into: group I (62 patients with SCA/Sß^0^ thalassemia); and group II (23 patients with SC hemoglobinopathy/Sß^+^ thalassemia). TCD was performed and reviewed by a single investigator using Doppler ultrasonography with a 2 MHz transducer, in accordance with the Stroke Prevention Trial in Sickle Cell Anemia (STOP) protocol. The hematological parameters evaluated were: hematocrit, hemoglobin, reticulocytes, leukocytes, platelets and fetal hemoglobin. Univariate analysis was performed and Pearson's coefficient was calculated for hematological parameters and TAMM velocities (P < 0.05).

**RESULTS:**

TAMM velocities were 137 ± 28 and 103 ± 19 cm/s in groups I and II, respectively, and correlated negatively with hematocrit and hemoglobin in group I. There was one abnormal result (1.6%) and five conditional results (8.1%) in group I. All results were normal in group II. Middle cerebral arteries were the only vessels affected.

**CONCLUSION:**

There was a low prevalence of abnormal Doppler results in patients with sickle-cell disease. Time-average maximum mean velocity was significantly different between the genotypes and correlated with hematological characteristics.

## RESUMO

**CONTEXTO E OBJETIVO:**

Doppler transcraniano (DTC) detecta risco de acidente vascular cerebral (AVC) em crianças com anemia falciforme (AF). O objetivo foi avaliar os resultados ao DTC nos diferentes genótipos da doença falciforme (DF) e correlacionar a velocidade média-máxima (VMMáx) às características hematológicas.

**TIPO DE ESTUDO E LOCAL:**

Estudo transversal analítico realizado no setor de Hematopediatria da Universidade Federal de São Paulo.

**MÉTODOS:**

85 pacientes com DF, 2-18 anos, ambos os sexos, foram avaliados e divididos em: grupo I (62 com AF ou Sß^0^ talassemia); e grupo II (23 com hemoglobinopatia SC ou Sß^+^ talassemia). DTC foi realizado e revisado por um único investigador usando um aparelho de ultrassonografia Doppler com transdutor de 2MHz, conforme critérios do protocolo STOP (Stroke Prevention Trial in Sickle Cell Anemia). As variáveis hematológicas avaliadas foram: hematócrito, hemoglobina, reticulócitos, leucócitos, plaquetas, hemoglobina fetal. Análise univariada e coeficiente de Pearson calculados para parâmetros hematológicos e VMMáx, P < 0,05.

**RESULTADOS:**

As média das VMMáx foram de 137 ± 28 cm/s e 103 ± 19 cm/s nos grupos I e II, respectivamente. Houve correlação negativa da VMMáx com hematócrito e hemoglobina no grupo I. Houve um (1,6%) resultado anormal e 5 (8,1%) condicionais no grupo I; no grupo II, todos estavam normais. Artérias cerebrais médias foram as únicas acometidas.

**CONCLUSÃO:**

Houve baixa prevalência de resultados anormais ao DTC em pacientes com DF. A VMMáx foi significativamente diferente entre os genótipos da DF e apresentou correlação com variáveis hematológicas.

## INTRODUCTION

Sickle cell disease is a hemoglobinopathy characterized by the presence of more than 50% hemoglobin S on hemoglobin electrophoresis. The homozygote SS form is known as sickle cell anemia. Hemoglobin S can occur in association with other anomalous types of hemoglobin, and among these are hemoglobin C and thalassemia beta, thus forming different genotypes of sickle cell disease: hemoglobin SC disease and S-beta thalassemia.^1^

It has been estimated that more than seven million people have hemoglobin S in Brazil, and that more than 25,000 to 30,000 people have the homozygote form, with more than 3,500 new cases born every year.^1,2^

In the S-beta globin gene, a normal codon (GAG) is replaced by another (GTG), thus resulting in exchanging the sixth amino acid of the beta globin. This exchange of glutamic acid for valine causes polymerization of hemoglobin S, when exposed to media with low oxygen tension, which leads to sickling of red blood cells. This vaso-occlusive process is responsible for most of the clinical manifestations of sickle cell disease.^3,4^

Stroke is one of the most feared clinical complications from this disease because of its high morbidity-mortality.^5^ In children and adolescents, the most common form is ischemic stroke, and around 5-10% of patients with sickle cell anemia present stroke by the age of 20 years.^3,5,6^ The physiopathology of ischemic stroke differs from the mechanisms of the other complications of the disease. In autopsy studies, arteriography and other diagnostic methods, it has been observed that ischemic stroke in cases of sickle cell anemia is caused by hypertrophy of the intimal layer of the intracranial arteries, with proliferation of fibroblasts and smooth muscle of blood vessels.^3,7,8^ Transcranial Doppler enables early detection of arterial abnormalities in individuals with sickle cell anemia, which is extremely important for instituting primary prevention.^9-14^

The normal blood flow velocities obtained through transcranial Doppler are influenced by several factors, of which the main three determinants are the difference in pressure gradient along the vessel, the vessel length and cross-sectional area (caliber) and the blood viscosity. The hematocrit level and leukocyte and platelet counts influence blood viscosity and thus may have a bearing on blood flow velocity.^15-20^

Adams et al. conducted several studies using transcranial Doppler among individuals with sickle cell anemia to demonstrate the usefulness of this examination for evaluating stroke risk. They defined normal blood flow velocity as values of up to 170 cm/s, intermediate values as 170 to 200 cm/s and values greater than 200 cm/s as critical, with a high risk of developing stroke.^8,18,19,21^ Among these patients, if they had abnormal results from two Doppler examinations, they received a transfusion for primary stroke prevention.^9^ Blood transfusion was used because it is highly effective in reducing the risk of recurrence stroke in sickle cell anemia.^7^

The clinical picture of sickle cell disease is very variable, and hematocrit levels, leukocyte counts and hemoglobin S and fetal hemoglobin percentages correlate with the clinical severity of these patients’ conditions.^22^ In this light, and given that these parameters have been little studied in relation to other genotypes of sickle cell disease, our aim in this study was to evaluate the results from transcranial Doppler among patients with sickle cell disease who were being followed at the Pediatric Hematology Outpatient Clinic of Universidade Federal de São Paulo (Unifesp), correlating the time-averaged maximum mean velocity obtained through transcranial Doppler with the different genotypes of sickle cell disease and the hematological characteristics.

## OBJECTIVE

To evaluate the results from transcranial Doppler among patients with sickle cell disease and correlate the time-averaged maximum mean velocity obtained through transcranial Doppler with the different genotypes of sickle cell disease and the hematological characteristics.

## METHODS

This was a single-center cross-sectional study on all patients with sickle cell disease between the ages of 2 and 18 years who came to the Pediatric Hematology Outpatient Clinic of Unifesp in 2004 and 2005.

The inclusion criteria were that the patients needed to have no previous clinical diagnosis of stroke and no acute crises.

Patients were excluded if they did not cooperate with transcranial Doppler examination, if they did not agree to undergo the examination, if they were using hydroxyurea or if they had received red blood cell transfusions over the preceding three months.

This study was approved by the Research Ethics Committee of Unifesp (no. 149/03). An informed consent statement was obtained from all the adults responsible for the patients participating in the study.

All the transcranial Doppler examinations were performed by the same professional, who had been trained to perform transcranial Doppler (Silva GS), using the Nicolet EME-Companion TC2000 apparatus with a 2 MHz transducer and following the criteria of the STOP (Stroke Prevention Trial in Sickle Cell Anemia) study.^9^ From this examination, the time-averaged maximum mean velocities were determined every 2 mm along the following arteries: bilateral internal carotid arteries, bilateral anterior cerebral arteries, anterior-middle cerebral artery bifurcations, middle cerebral arteries, bilateral posterior cerebral arteries, bilateral vertebral arteries and basilar artery. The highest value from the right or left middle cerebral arteries, bilateral internal carotid arteries or anterior-middle cerebral artery bifurcations was taken as the time-averaged maximum mean velocity for each patient and was used in the data analysis. When the time-averaged maximum mean velocity result was conditional or abnormal, the examinations were repeated until two consecutive abnormal results were obtained.

The blood tests performed were total hemoglobin and hematocrit assays; leukocyte and platelet counts, performed in an electronic counter (Coulter model Ssr or Coulter model T-890); hemoglobin S assay by means of hemoglobin electrophoresis on cellulose acetate with tris-EDTA-borate buffer (pH 8.6); and fetal hemoglobin assay, quantified by means of alkaline denaturation.

The patients were divided into two groups, in accordance with the clinical and hematological differences already recognized in the literature. One group was composed of patients with the homozygote form and Sβ^0^ thalassemia (group I), and the other was composed of patients with SC hemoglobinopathy and Sβ^+^ thalassemia (group II).

The results were described through calculations of means and standard deviations for variables with normal distribution. Hematological parameters predictive of time-averaged maximum mean velocities were investigated by means of univariate analysis. Pearson's coefficient was calculated to evaluate correlations between the time-averaged maximum mean velocity and the hemoglobin S percentage, fetal hemoglobin percentage, hemoglobin level, hematocrit percentage, platelet count, leukocyte count and reticulocyte percentage. The Statistical Package for the Social Sciences (SPSS) 10.0 software (SPSS Inc., Chicago, United States) was used for the statistical analyses. P values < 0.05 were taken to be statistically significant.

## RESULTS

By the end of the study period, 85 patients with sickle cell disease had been evaluated. Group I was composed of 62 patients and group II was composed of 23 patients (Table 1). There were no statistically significant differences between the groups with regard to age and sex.

**Table 1.** Demographic characteristics of the patients with sickle cell disease (groups I and II) who underwent transcranial Doppler examination

Genotypes	Sickle cell disease genotype			
	SS	Sβ^0^ thal	SC	Sβ^+^ thal
n (%)	57 (67.1%)	5 (5.9%)	19 (22.3%)	4 (4.7%)
	Group I		Group II	
n (%)	62 (73%)		23 (27%)	
Age*	8 y 11 m ± 4 y 4 m (2 y 4 m to 17 y 5 m)		7 y 6 m ± 4 y 0 m (2 y 7 m to 16 y 2 m)	
Male sex n (%)	32 (51.6%)		13 (56.5%)	

SS = sickle cell anemia (homozygote);Sβ^0^ thal = S-beta^0^ thalassemia; SC = sickle cell hemoglobinopathy; Sβ^+^ thal = S-beta+ thalassemia; n = number of patients; y = years; m = months.

( ) = range of values found;

* mean and standard deviation. Student's t test

We observed statistically significant differences between the two groups for all the hematological parameters (Table 2).

**Table 2.** Comparison between hematological parameters and time-averaged maximum mean velocity obtained using transcranial Doppler in groups I and II

	Patients		
	Group I SS and Sβ^0^ (n = 62)	Group II SC and Sβ^+^ (n = 23)	P
Hb (g/dl)*	8.0 ± 1.1 (5.7-11.4)	10.4 ± 1.1 (7.7-12.0)	P < 0.01
Htc (%)*	23.0 ± 4.0 (16.0-34.0)	31.0 ± 3.0 (23.0-36.0)	P < 0.01
HbF (%)*	13.3 ± 10.0 (0.0-45.9)	4.8 ± 5.7 (0.0-18.7)	P = 0.03
Platelet count (× 10^−3^/mm^3^)*	462129 ± 162443 (78000-986000)	316435 ± 172510 (97000-787000)	P < 0.01
Leukocyte count (× 10^−3^/mm^3^)*	14000 ± 4594 (6100-31200)	9765 ± 3072 (5000-15200)	P = 0.03
Reticulocytes (%)*	8.6 ± 6.4 (0.4-28.9)	2.3 ± 1.7 (0.3-6.0)	P < 0.01
TAMM (cm/s)*	137.0 ± 28.0 (85.0-240.0)	103.0 ± 19.0 (83.0-145.0)	P = 0.01

Sβ^0^ thal = S-beta^0^ thalassemia; Sβ^+^ thal = S-beta+ thalassemia; Hb = hemoglobin; Htc = hematocrit; HbF = fetal hemoglobin; TAMM = time-averaged maximum mean velocity from transcranial Doppler.

( ) = range of values found;

* mean and standard deviation; Student's t test. Statistical significance: P < 0.05.

Group I presented a negative correlation between the time-averaged maximum mean velocity and the hematocrit percentage and between the time-averaged maximum mean velocity and the hemoglobin level. Group II presented a positive correlation between the time-averaged maximum mean velocity and the reticulocyte count and a negative correlation between the time-averaged maximum mean velocity and the fetal hemoglobin percentage (Table 3).

**Table 3.** Pearson correlation coefficient between mean maximum velocity relating to transcranial Doppler and hematological parameters, for groups I and II

	Pearson coefficient	
Hematological parameters	Group I (n = 62)	Group II (n = 23)
Hematocrit	- 0.38*	- 0.29
Hemoglobin	- 0.32*	- 0.24
Reticulocyte percentage	0.09	0.49*
Platelet count	Platelet count	0.18
Leukocyte count	0.14	0.22
Hemoglobin S percentage	0.18	0.17
Hemoglobin F percentage	- 0.16	- 0.35

Pearson's correlation coefficient results were classified as follows: Negative value: inverse correlation; Positive value: positive correlation; None: 0.00; Weak: between 0.01 and 0.29; Medium: between 0.30 and 0.59; Strong: between 0.60 and 0.89; Very strong: between 0.90 and 0.99; and Perfect: 1.00.

* Statistical significance: P < 0.01.

Only one patient in group I presented an abnormal velocity (1.6%). This patient with abnormal velocity had a conditional result (190 cm/s) in the first transcranial Doppler that was performed and, in the repetitions, presented abnormal velocity in two consecutive examinations (time-averaged maximum mean velocities of 220 cm/s and 240 cm/s). Five patients (8.1%) in group I presented conditional time-averaged maximum mean velocities (Table 4).

**Table 4.** Frequencies of time-averaged maximum mean velocity result categories from transcranial Doppler on two groups of patients with sickle cell disease

TAMM results from TCD			
	Normal n (%)	Conditional n (%)	Abnormal n (%)
Group I (SS and Sβ^0^) n = 62	55 (90.3)	5 (8.1)	1 (1.6)
Group II (SC and Sβ^+^) n = 23	23 (100.0)	0 (0.0)	0 (0.0)

TCD = transcranial Doppler; TAMM = time-averaged maximum mean velocity. normal TAMM: < 170 cm/s; conditional TAMM: between 170 and 200 cm/s; abnormal TAMM: > 200 cm/s.

It was observed that the arteries affected in these patients were the right or left middle cerebral artery in all the cases, both in the patient with abnormal and in the patients with conditional time-averaged maximum mean velocities.

## DISCUSSION

The use of transcranial Doppler has become an important tool for stroke prevention among patients with sickle cell anemia. Stroke occurs four times more often among sickle cell anemia patients than among patients with SC hemoglobinopathy or Sβ thalassemia.^5^

In the STOP study, which was carried out in 1998 and is the biggest study on transcranial Doppler in relation to patients with sickle cell anemia within the pediatric age group, the proportion of abnormal results was 9.7%.^9^ Thus, the results differed from those of the present study, in which only one patient with a time-averaged maximum mean velocity greater than 200 cm/s was found, representing 1.6%. This finding is in agreement with the results from other studies conducted in Brazil, which also found lower frequencies of abnormal results.^23-25^

In Sergipe, in 2005, Melo et al. gathered data on 34 patients with sickle cell anemia, aged less than 18 years, and compared them with 80 controls. Among the results from the patients, none of them (0.0%) presented abnormal time-averaged maximum mean velocity and four (11.7%) presented conditional results.^24^

In São Paulo, Park et al. evaluated 77 patients with sickle cell disease, aged between 2 and 16 years. They found two patients (2.6%) with abnormal time-averaged maximum mean velocity and 11 (14.3%) with conditional results.^23^

In Minas Gerais, in 2008, Silva et al. evaluated 153 children with sickle cell anemia, aged 2 to 16 years. They found seven patients (4.6%) with abnormal time-averaged maximum mean velocity and 16 (10.4%) with conditional results.^25^

In 1999, Kinney et al.^22^ investigated correlations of risk factors for stroke among patients with sickle cell anemia and found a positive correlation with the Senegal haplotype. In Brazil, the most common haplotype is Bantu, which may be a factor contributing towards this difference. The presence of alpha thalassemia, adherence molecules and thrombophilic factors such as hyperhomocysteinemia or factor V Leiden have been suggested as other likely factors involved in stroke risk.^22,26-30^

Group II did not present any abnormal or conditional result from transcranial Doppler, probably because of the lower severity of these patients’ condition. So far, there are no data in the literature indicating screening for stroke risk using transcranial Doppler among other genotypes of sickle cell disease. In 1990, Adams et al. evaluated patients with sickle cell anemia and SC hemoglobinopathy, although only 10 SC hemoglobinopathy patients were included in the sample (190 patients) and they were analyzed together. In other studies, only patients with sickle cell anemia were included.^9,10,15,21^

In agreement with several other studies, there was a negative correlation in group I between the time-averaged maximum mean velocities and the hematocrit percentages and hemoglobin levels.^15-17,19,20,23-25^ No comparison with the literature could be made with regard to the correlation that was found in group II between time-averaged maximum mean velocities and the reticulocyte percentages and fetal hemoglobin levels, since no such data is available in the literature.

There was a statistically significant difference in the time-averaged maximum mean velocities between the groups, such that it was greater in group I. This was probably because of the lower hemoglobin levels and lower hematocrit percentages in this group (P < 0.01).

Abnormalities were only observed in the middle cerebral artery, in both cerebral hemispheres, and these were found both in the patient with abnormal time-averaged maximum mean velocities and in the patients with conditional velocities. In 1992, Adams found that around 60% of the middle cerebral arteries were affected, and that the internal carotid arteries and anterior cerebral arteries were also affected.^8,21^

Our results emphasize the importance of performing transcranial Doppler on cases of sickle cell anemia. National studies in Brazil involving the use of transcranial Doppler on large numbers of patients with sickle cell disease are needed, given that some findings differ from data in the literature. It is still uncertain whether there is a determining factor that would explain such differences, or whether the sample size would explain these findings.

Transcranial Doppler does not seem to be a priority examination in relation to the other genotypes of sickle cell disease. Longitudinal follow-up studies on patients with different sickle cell genotypes and their respective transcranial Doppler findings are needed to confirm this hypothesis.

## CONCLUSION

There was a low prevalence of abnormal Doppler results in patients with sickle-cell disease. Time-average maximum mean velocity was significantly different between the genotypes and correlated with hematological characteristics.
